# In vivo evaluation of facial papule dermatoses with reflectance confocal microscopy in children

**DOI:** 10.1111/srt.13170

**Published:** 2022-06-21

**Authors:** Lixin Chen, Ying Wang, Xibo Gao, Bei Qin, Jia Lian, Min Ren, Wanxing Zhang, Ran Wei, Qinfeng Li

**Affiliations:** ^1^ Department of Dermatology Tianjin Children's Hospital Tianjin China

**Keywords:** children, facial papule dermatoses, juvenile xanthogranuloma, keratosis pilaris, milia, molluscum contagiosum, reflectance confocal microscopy, seborrheic keratosis, verruca plana

## Abstract

**Background:**

Molluscum contagiosum (MC), milia, keratosis pilaris (KP), verruca plana (VP), seborrheic keratosis (SK), and juvenile xanthogranuloma (JXG) are common papule dermatoses on the face of children that have a similar appearance. In vivo evaluation of facial papule dermatoses with reflectance confocal microscopy (RCM) is helpful in the diagnosis of these ambiguous lesions in children. The purpose of this study was to clarify the RCM characteristics of MC, milia, KP, VP, SK, and JXG and explore the clinical application value of RCM for these common facial papule dermatoses.

**Methods:**

We recruited 113 patients referred for unequivocal facial papule dermatosis, including 21 patients with MC, 17 patients with milia, 19 patients with KP, 36 patients with VP, 8 patients with SK, and 12 patients with JXG. We evaluated the characteristics and distinguishing features of the six kinds of facial papule dermatoses using RCM.

**Results:**

The main RCM features of the six dermatoses included a well‐demarcated border of the lesion area. MC, milia and KP all manifested cyst‐like structures, and their distinguishing features were the location of the cystic structures and the refractive index of the contents. Although VP, SK, and JXG did not have obvious cystoid structures, VP was typically characterized by uniformly distributed petal‐like structures with a medium‐to‐high refractive index in the epidermis. With regard to SK, the characteristic features were an obviously thickened epidermis and cobblestone‐like structures. JXG was mainly characterized by multiple large round and ovoid cells with a foamy cytoplasm, and discoid‐shaped multinucleated large cells were diffusely distributed in the dermis.

**Conclusion:**

RCM allows the real‐time visualization of major key diagnostic and distinguishing features of common facial papule dermatoses in children, including MC, milia, KP, VP, SK, and JXG.

## 1 INTRODUCTION

Molluscum contagiosum (MC), milia, keratosis pilaris (KP), verruca plana (VP), seborrheic keratosis (SK), and juvenile xanthogranuloma (JXG) are common dermatoses on the face of children and have similar papule appearances. Because of the patients’ age and the primarily facial locations of the lesions, the patients and parents are unwilling to undergo biopsy, making noninvasive examinations with reflectance confocal microscopy (RCM) necessary. RCM is a powerful, noninvasive imaging technique that provides high‐resolution cellular imaging of the epidermis and superficial dermis.^1^ The aims of this study were to systematically summarize the RCM features of MC, milia, KP, VP, SK, and JXG and determine the distinguishing features of these six skin conditions.

## 2 MATERIALS AND METHODS

### 2.1 Patients

**FIGURE 1:**
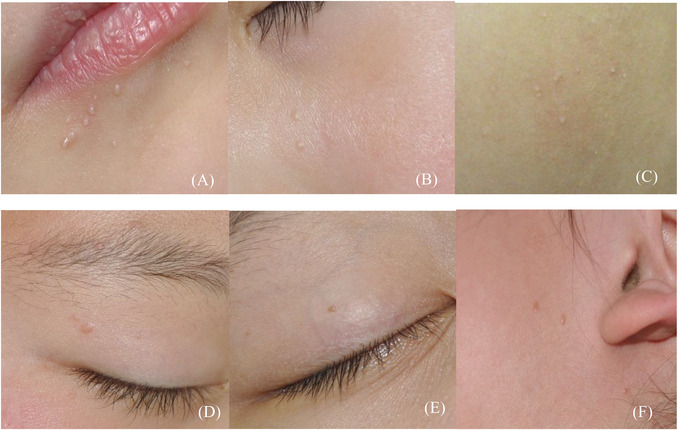
Clinical presentation of six facial papule dermatoses in children: (A) molluscum contagiosum (MC): multiple, flesh‐colored and translucent papules; (B) milia: elevated, and skin‐colored papules; (C) keratosis pilaris (KP): follicular, skin‐colored, or light brown papules; (D) verruca plana (VP): slightly elevated, flat‐topped, and skin‐colored papules; (E) seborrheic keratosis (SK): discrete, gray–brown, and flat papules; (F) juvenile xanthogranuloma (JXG): multiple, dome‐shaped, and yellow–red papules

A total of 113 patients (21 with MC, 17 with milia, 19 with KP, 36 with VP, 8 with SK, and 12 with JXG) were included in the study (Figure 1). The patients were recruited from the Outpatient Clinic at Tianjin Children's Hospital and referred for RCM examination. All patients were aged 1–11 years. The symptoms of the skin conditions had occurred for an average age of 2.65 months (ranging from 0.5 to 9 months). All procedures performed in this study involving human participants were in accordance with the ethical standards of the National Research Committee.

The inclusion criteria were as follows: (1) solitary papule dermatosis with a definitive diagnosis made by two independent, clinically experienced dermatologists according to clinical presentations and dermoscopic findings; (2) no topical or systemic treatment before RCM examination; and (3) consent to participate in the study. The exclusion criterion was an inability to undergo the RCM examination.

### 2.2 In vivo RCM

All patients included in the study were examined using a commercially available RCM system, a VivaScope 1500 (Lucid Inc., Rochester, NY, USA). The system uses an infrared 830‐nm diode laser operating at a power of less than 20 mW at the tissue level. A ×30 water‐immersion objective lens with a numerical aperture of 0.9 was used, and pure water (refractive index = 1.33) was used as an immersion medium. The probe was routinely disinfected with 75% alcohol before and after each examination.

## 3 RESULTS

### 3.1 MC, milia, and KP

**FIGURE 2:**
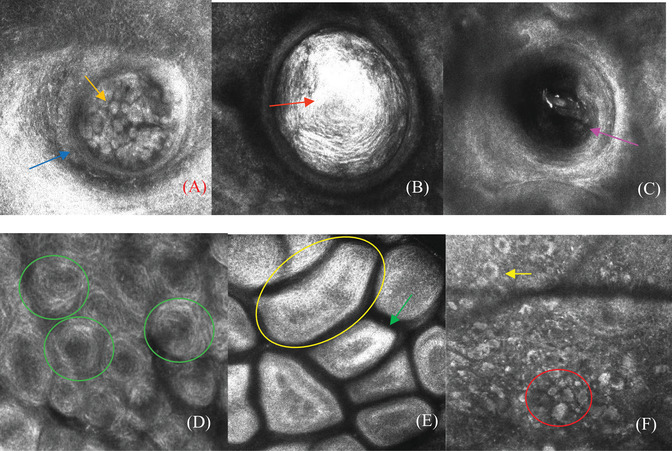
Reflectance confocal microscopy (RCM) characteristics of six facial papule dermatoses in children: (A) in molluscum contagiosum (MC), the cyst‐like areas are arranged in a dike‐like structure and filled with medium refractive, roundish structures (orange arrow), and the cyst wall is remarkable (blue arrow); (B) in milia, an oval, amorphous, and highly refractive substance is observed within the cyst‐like structure (red arrow), and the cyst wall is inconspicuous; (C) in keratosis pilaris (KP), medium‐to‐low refractive keratinoid are visible in the expanded infundibulum of the hair follicle (pink arrow), and there is no “cyst wall”; (D) in verruca plana (VP), medium‐to‐highly refractive petal‐like structures are found in the epidermis (green oval); (E) in seborrheic keratosis (SK), medium‐to‐highly refractive cobblestone‐like structures (yellow oval) and enlarged and bright dermal papillary rings are observed (green arrow); (F) in juvenile xanthogranuloma (JXG), multiple large round‐to‐ovoid cells (red oval) and discoid‐shaped multinucleated large cells (yellow arrow) are seen in the dermis

MC, milia, and KP were characterized by cyst‐like structures that were round to oval in shape, with a well‐demarcated border. For MC, the cyst‐like areas were located in the epidermis and were arranged in a dike‐like structure. The cyst wall was remarkable (21/21, 100%). Sometimes, the structures were separated into hyporefractive lobules by septa (7/21, 33.3%) and filled with medium‐refractive roundish structures. As the depth of the imaging increased, the refraction of the roundish structures tended to slowly decrease (Figure 2A). In milia, no obvious abnormality in the epidermis was observed. Confocal imaging of the milia papules showed the presence of cavity‐like structures in the dermis. The cyst wall of these structures was inconspicuous, and a round/oval, amorphous, highly refractive substance could be seen within (17/17, 100%). The refractive index did not change significantly with the depth of imaging, and no septa were observed (Figure 2B). For KP, the “cystic cavity” was actually the expanded infundibulum of a hair follicle, with no “cyst wall.” Keratinoid containing a medium‐to‐low refractive substance could be seen (19/19, 100%), and the refraction tended to significantly decrease with the depth of imaging (Figure 2C). A linear medium‐to‐highly refractive hair shaft‐like structure was occasionally visible in the “cystic areas” (14/19, 73.7%) (Table 1).

**TABLE 1:** Key RCM features of MC, milia, and KP

Condition	Location	Cyst wall	Contents
MC	Starting from epidermis; septa (+)	Remarkable; dike‐like	Medium refractive, roundish structures
Milia	Dermis; septa (−)	Inconspicuous	Amorphous highly refractive substance
KP	Infundibulum of a hair follicle	–	Medium‐to‐low refractive keratinoid; medium‐to‐highly refractive hair‐shaft‐like structure

Abbreviations: KP, keratosis pilaris; MC, molluscum contagiosum; RCM, reflectance confocal microscopy.

### 3.2 VP, SK, and JXG

There was no obvious cystic structure in the RCM images of VP, SK, and JXG. In VP lesions, confocal imaging revealed the presence of uniformly distributed medium–highly refractive petal‐like structures in the stratum granulosum and the stratum spinosum (27/36, 75%), and low‐refractive areas could be seen among the petal‐like structures (36/36, 100%) (Figure 2D). For SK lesions, the epidermis was obviously thickened and distributed as cobblestone‐like structures. Low‐refractive areas between the cobblestone‐like structures were marked, and enlarged and bright dermal papillary rings were detected at the dermoepidermal junction (8/8, 100%) (Figure 2E). For JXG, epidermal examination revealed a normal honeycomb pattern or slight atrophy. In the dermis, multiple large round‐to‐ovoid cells with foamy cytoplasm were diffusely distributed, and discoid‐shaped multinucleated large cells with a peripheral bright ring were also observed (12/12, 100%) (Figure 2F, Table 2).

**TABLE 2:** Key RCM features of VP, SK, and JXG

Condition	Epidermis	Dermoepidermal junction/dermis
VP	Medium‐to‐highly refractive petal‐like structures	Dilated blood vessels and inflammatory cells
SK	Medium‐to‐highly refractive cobblestone‐like structures	Enlarged and bright dermal papillary rings
JXG	Normal or slightly atrophic	Multiple large round‐to‐ovoid cells; discoid‐shaped multinucleated large cells

Abbreviations: JXG, juvenile xanthogranuloma; RCM, reflectance confocal microscopy; SK, seborrheic keratosis; VP, verruca plana.

## 4 DISCUSSION

Pediatric patients are a special group in whom invasive procedures such as biopsies are not always possible. RCM is a real‐time, repeatable, complementary diagnostic tool that allows noninvasive imaging to a depth of 200–300 μm, with resolution comparable to histopathology, and provides an objective basis for the diagnosis of conditions with similar clinical manifestations.^1^, ^2^

The clinical manifestations of MC, milia, and KP are similar isolated papules that are yellowish‐white or skin colored (Figure 1). MC is a common skin infection caused by a poxvirus and is generally observed in children. MC lesions typically appear as single or multiple, small (2–5 mm), flesh‐colored, and translucent papules with a characteristic central umbilication. Milia are elevated papules that are skin‐colored or yellow, approximately 2–4 mm in size, and appear on the face. The clinical manifestations of KP are follicular papules that are skin‐colored or light brown and 1–2 mm in size. On RCM, the lesions of MC, milia, and KP all appeared as cyst‐like structures, but the location, wall, and contents of these lesions were completely different. The crater‐like cyst structures of MC were located in the epidermis, and the cysts were filled with medium‐refractive roundish structures, whereas the “cystic structures” of milia were located in the dermis and were filled with irregularly distributed, highly refractive materials. The “cystic cavity” structure of KP was actually the expanded infundibulum of a hair follicle, and a medium‐refractive material and a highly refractive hair‐like structure were seen inside (Figure 2, Table 1).

VP, SK, and JXG can clinically manifest as similar skin‐colored or yellowish papules (Figure 1). VP is a viral infectious skin condition, revealing slightly elevated, flat‐topped, skin‐colored, or brown papules measuring 1–4 mm on the face. In 2008, González^3^ reported the presence of multiple highly refractile round structures within VP lesions. Liu^4^ further reported the main characteristics of VP and named the round structures petal‐like structures, and experts^5^, ^6^, ^7^ reached a consensus that the uniform distribution of the highly refractive petal‐like structures is the main feature of VP. SK consists of ubiquitous, generally benign skin tumors that exhibit high clinical variability. Age is a known risk factor, as SK is highly prevalent in older populations and seems to increase in prevalence with age but can also be seen in children. It usually occurs on the face, with skin‐colored or gray–brown, flat papules. In children, SK is easily confused with VP. On RCM, SK‐specific characteristics include cerebriform‐shaped, abnormal dermal papilla, which have been proven to be very valuable in adult^2^, ^4^, ^8^ but not pediatric patients. We found that the RCM features of SK in children were cobblestone‐like structures and enlarged and bright dermal papillary rings at the dermoepidermal interface. JXG is a benign, proliferative disorder of histiocytic cells belonging to the group of non‐Langerhans cell histiocytoses. JXG occurs predominantly in infants and children and, in this population, manifests as a solitary or multiple, dome‐shaped yellow–red papule/nodules, with a maximum diameter of 1 cm upon physical examination. Diagnostic skin biopsy reveals Touton giant cells, the classic foamy histiocytes typically seen in JXG. RCM examination revealed that the dermal papillae were filled with multiple large ovoid cells with foamy cytoplasm and discoid‐shaped multinucleated large cells. The large ovoid cells with foamy cytoplasm correspond to the foamy histiocytes seen on histopathology, and the multinucleated large cells correspond to Touton giant cells.^9^ The previously mentioned results demonstrated that there is a high degree of correlation between the observed RCM features of JXG and its histologic findings (Figure 2, Table 2).

## 5 CONCLUSION

MC, milia, KP, VP, SK, and JXG all clinically manifest as similar skin‐colored or yellowish papules but have differentiating RCM characteristics in specific skin layers. These results indicate the potential value of RCM imaging for the differential diagnosis of MC, milia, KP, VP, SK, and JXG, and to a certain extent, the use of RCM avoids the need for invasive biopsy.

## CONFLICT OF INTEREST

The authors declare no conflict of interest.

6

## Data Availability

The data that support the findings of this study are available from the corresponding author upon reasonable request.
